# Microscopic distribution of taxanes in freeze-fixed stems of *Taxus cuspidata*

**DOI:** 10.3389/fchem.2024.1437141

**Published:** 2024-08-12

**Authors:** Qinyue Gong, Dan Aoki, Masato Yoshida, Kazuhiko Fukushima

**Affiliations:** Graduate School of Bioagricultural Sciences, Nagoya University, Nagoya, Aichi, Japan

**Keywords:** *Taxus cuspidata*, *Taxaceae*, cryo-TOF-SIMS/SEM, mass spectrometry imaging, taxanes, paclitaxel

## Abstract

**Introduction:**

*Taxus* species contain the anticancer alkaloid paclitaxel, as well as other taxanes similar in structure and potentially in effect to paclitaxel. Tissue-specific distribution patterns and seasonal variations of taxanes in some *Taxus* species have been reported; however, it is still under-presented for the taxanes in *Taxus cuspidata*.

**Methods:**

The radial distributions of eight taxanes in the transverse surface of freeze-fixed *T. cuspidata* stems from the late summer and the spring seasons were investigated by cryo-time-of-flight secondary ion mass spectrometry and scanning electron microscopy (cryo-TOF-SIMS/SEM) visualization and liquid chromatography-mass spectrometry (LC-MS) quantitative analysis. By optical microscopic observation, seasonal differences in the amounts and distribution patterns of target taxanes were further characterized in specific tissues.

**Results and Discussion:**

The overall amount of taxanes was higher in the late summer than in the spring. Also, taxanes’ radial distribution was generally found at higher concentration in the phloem, the cambium and lower level in the periderm, the latest-forming xylem, with different taxanes showing several patterns with distinction between seasons, which were considered related to seasonal plant physiological behaviors. In addition, the distribution of baccatin III (BAC) was investigated at the cellular level, which was regarded in specific cells suggesting its transport in the radial and axial directions in the *T. cuspidata* stem. Characterizing the microscopic distribution of taxanes in the *T. cuspidata* stem is expected to play a role in the further study of their biosynthesis and *in planta* behaviors.

## 1 Introduction

*Taxus* species belonging to the family *Taxaceae* have been valued as significant medicinal plants for containing the alkaloid paclitaxel, one of the most important naturally occurring chemotherapy medications against cancer. In addition to paclitaxel, there is a considerable amount of other taxanes in *Taxus* species, such as cephalomannine (CE), baccatin III (BAC), and 10-deacetylbaccatin III (10-DAB), which were reported to be involved as the precursors in the putative biosynthesis pathway as well as the reported semi-synthetic process of paclitaxel (Guerra-Bubb et al., 2012; Howat et al., 2014; Zhang et al., 2023); and 7-epi-10-deacetyltaxol (EDT), 10-deacetyltaxol (10-DAT), 7-epi-taxol, 7-xylosyl-10-deacetyltaxol (7-xyl-10-DAT), which were reported to exert potential of cytotoxicity to tumor cells (Helson, 1993; Shang et al., 2011; Dang et al., 2017; Subban et al., 2017).

Paclitaxel accumulates at different levels among distinct *Taxus* species, one of the reasons for which was assumed to be due to the variation of its analogs, especially those involved in paclitaxel biosynthesis (Yu et al., 2018). Various taxanes have been successively isolated or determined from the leaves, seeds, and branches bark of *Taxus cuspidata* Siebold & Zucc. (Shi et al., 2000; Cao et al., 2006; Zhang et al., 2010; Ni et al., 2011; Gai et al., 2020). Some taxanes with structural similarity to paclitaxel were found to possess proximate biological effects as well (Shigemori & Kobayashi, 2004; Chakravarthi et al., 2013); however, a comprehensive understanding of the *in planta* effects of taxanes and biosynthetic pathways that involved taxanes in the local *Taxus* species in Japan, *T*. *cuspidata*, is still lacking.

Seasonal variations in the content of several taxanes in the stem of *Taxus baccata* (Glowniak et al., 1999) and *Taxus brevifolia* (Vance et al., 1994) have been reported. In addition, taxanes were found to exhibit tissue-specific distribution patterns in *Taxus media* (Soliman & Raizada, 2020; Yu et al., 2020) and *Taxus wallichiana* var. *mairei* (Yu et al., 2023). However, the taxane distribution at the cellular level is still under-elucidated. The spatial distribution of taxanes among seasons is considered to lay the foundation for revealing their biological roles and activities in *Taxus* plants, for which mass spectrometry imaging is considered an effective tool. In contrast to matrix-assisted laser desorption/ionization mass spectrometry (MALDI-MS), which is one of the most commonly used techniques, secondary ion mass spectrometry with a higher spatial resolution is particularly effective for obtaining localization information of target compounds at the cellular level (Aoki et al., 2022). The authors have developed an analytical system including a glove box (N_2_ environment, −20°C), a cryo-vacuum shuttle, a time-of-flight secondary ion mass spectrometer (TOF-SIMS), and a scanning electron microscope (SEM) (Kuroda et al., 2013; Masumi et al., 2014; Aoki et al., 2022), in which the plant sample could be prepared to obtain a fresh surface and transferred by the cryo-vacuum shuttle to TOF-SIMS and SEM for analysis while maintaining frozen and hydrated. With this system, the visualization of salicifoline in the freeze-fixed stems of *Magnolia kobus* (Okumura et al., 2017) and benzylisoquinoline alkaloids in the freeze-fixed stems of *Phellodendron amurense* (Gong et al., 2023) have been realized, which proved the possibility of achieving mapping of alkaloids in specific cellular tissues of plant samples with the losses of the target compounds minimized.

In this study, the radial distribution of eight taxanes was investigated in the transverse surface of freeze-fixed *T. cuspidata* stems from the late summer and the spring seasons by cryo-TOF-SIMS/SEM visualization and liquid chromatography-mass spectrometry (LC-MS) quantitative analysis. The seasonal differences in the amounts and distribution patterns of the target taxanes were further characterized in specific tissues by optical microscopy observation. Also, the distribution of one of the taxanes, BAC, as a representative intermediate in paclitaxel biosynthesis, was discussed at the cellular level with the possible biological activities *in planta*.

## 2 Results

### 2.1 Radial quantitative distributions of taxanes by LC-MS

**FIGURE 1:**
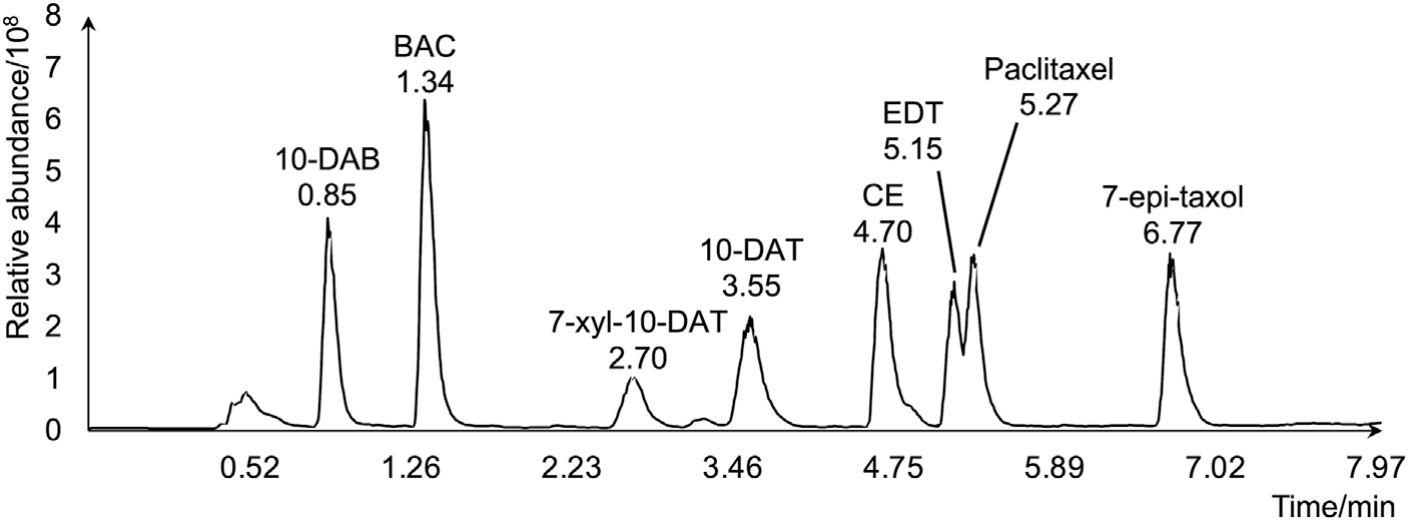
Total ion chromatogram of the eight target taxanes by LC-MS.

**FIGURE 2:**
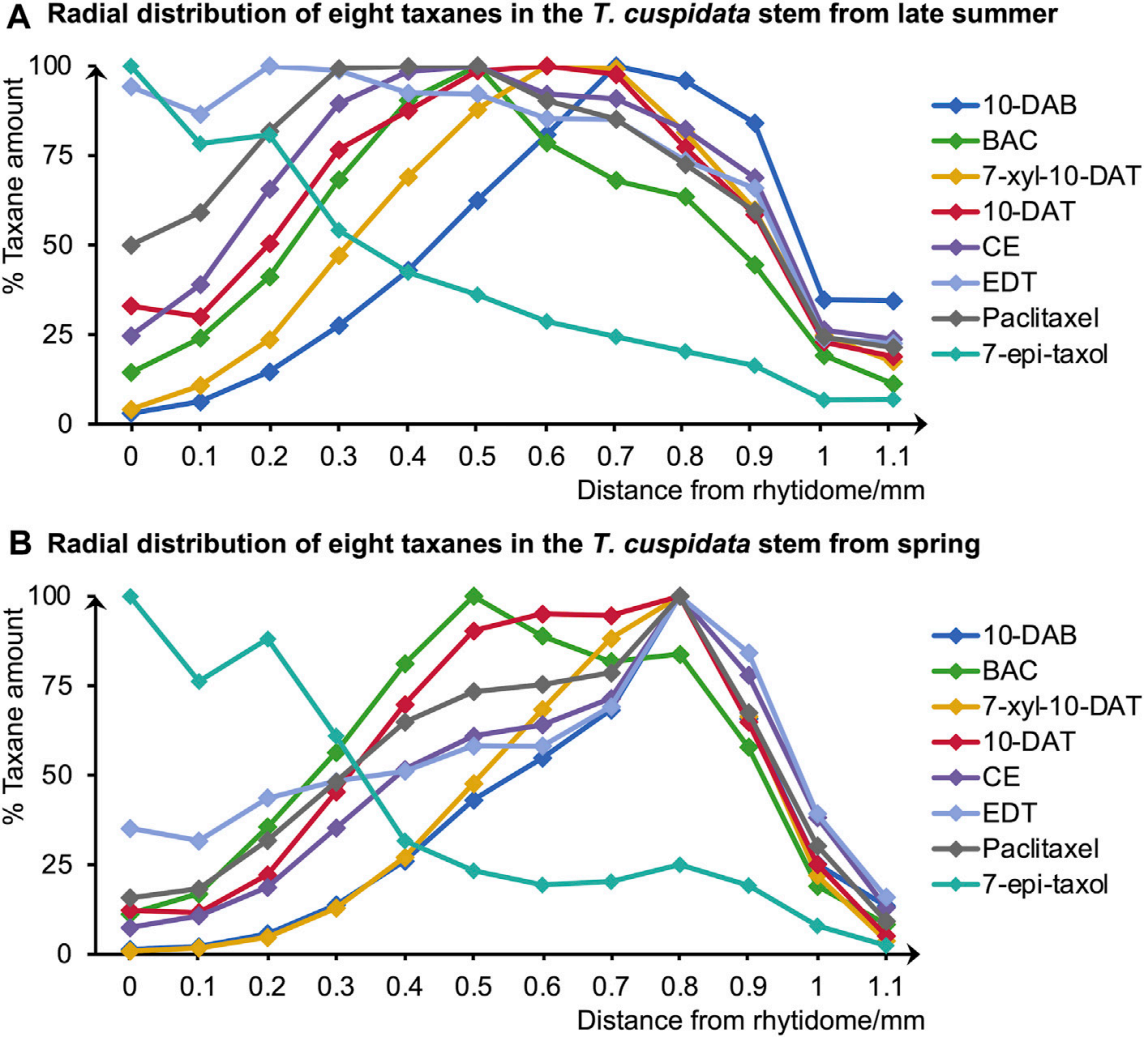
Radial distributions of the eight taxanes in **(A)** late summer and **(B)** spring *T. cuspidata* stems quantified by LC-MS using serial tangential sections (0–1.1 mm from the rhytidome). Means for each sample were obtained from three sets of measurements (n = 3) using different sample blocks from the same disk.

To evaluate the amounts and radial distributions of taxanes in *T. cuspidata*, 12 serial tangential sections of 100-μm thickness were obtained from the rhytidome to the latest-forming xylem from the freeze-fixed blocks of the *T. cuspidata* stems sampled in the end of September (late summer) and the end of the following May (spring). The obtained sections were extracted with 80% ethanol (EtOH) aqueous solution (aq.) and measured by LC-MS (Supplementary Figure S1). The total ion chromatogram of the taxane standards by LC-MS is shown in Figure 1, and the masses with errors of the detected taxane ions are shown in Supplementary Table S1. The relative contents of all eight taxanes in radial sections are aggregated separately by seasons (Figure 2), and the differential distributions of each target taxane between seasons are shown in Supplementary Figure S2.

According to the quantification results of the total amounts of the eight target taxanes, they were generally higher in the late summer than in the spring (Supplementary Figures S2). Besides, paclitaxel, CE, 10-DAT, and 7-xyl-10-DAT generally had higher amounts than BAC, 10-DAB, EDT, and 7-epi-taxol in both seasons.

**FIGURE 3:**
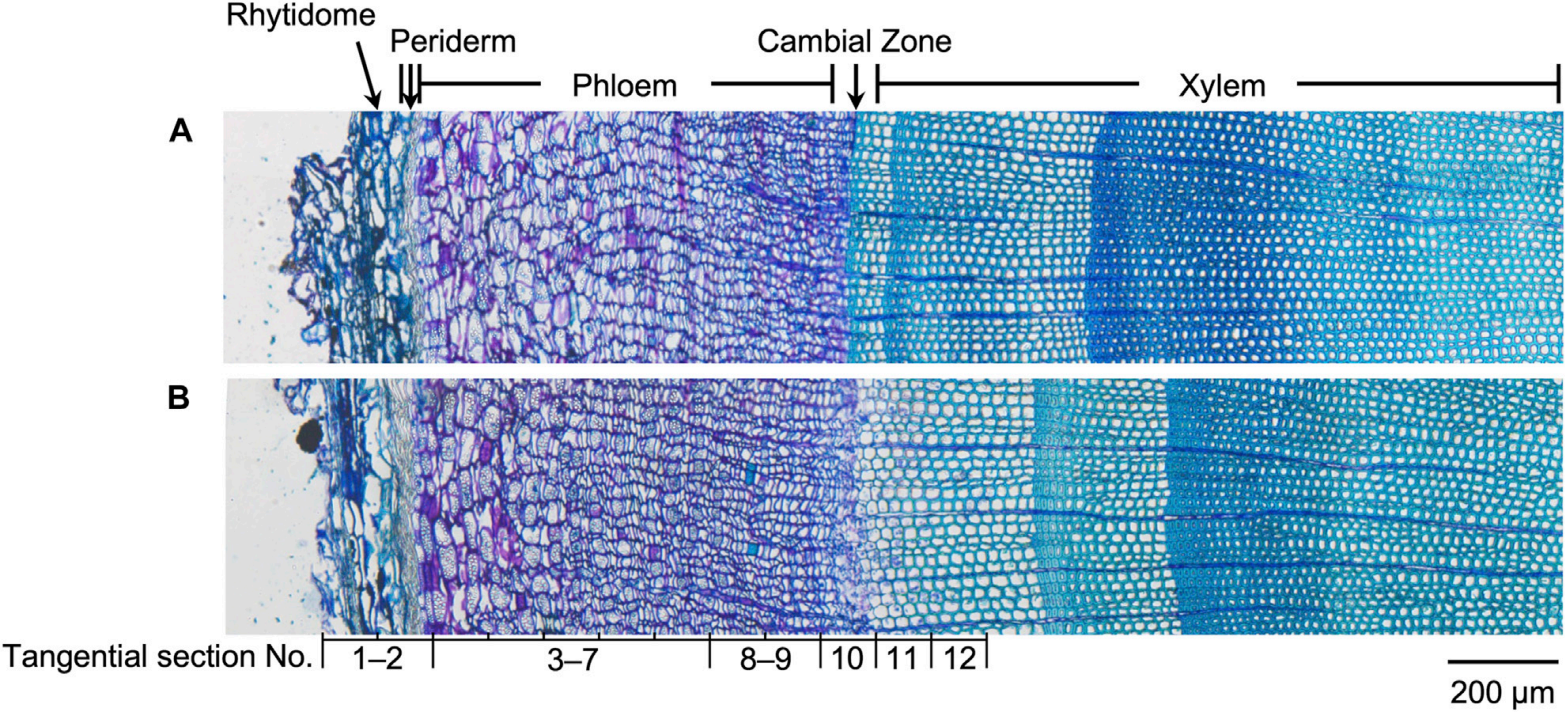
Optical microscopic images of toluidine blue stained sections containing rhytidome, periderm, phloem, cambial zone, and xylem obtained from freeze-fixed stems of *T. cuspidata* in **(A)** late summer and **(B)** spring. The tissue assignment of the tangential sections for quantification is shown by the section numbers correspondingly. The scale bar is 200 μm.

Since the major tissue division of blocks from the same disk is considered similar, the radial tissue assignment of the tangential sections can be defined by referring to the optical microscopic images with scales of the transverse sections from the same sample disks (Figure 3). Coarse tissue assignments are given as a reference (Table 1) to discuss the radial quantitative distributions of taxanes, based on which the presence of all eight taxanes from the periderm to the xylem was confirmed to be with higher concentrations in the phloem, the cambium, and lower levels in the periderm, the latest-forming xylem (Figure 2). 10-DAB and 7-xyl-10-DAT were found to have slightly different distributions in the late summer samples: both of them had a peak distribution in the phloem region, while 10-DAB showed a more concentrated distribution in the later-forming part. When it came to spring, the accumulation of 10-DAB seemed not significantly changed, while the accumulation of 7-xyl-10-DAT tended to be consistent with 10-DAB (Supplementary Figures S2A, B). Similar to the distribution of 7-xyl-10-DAT in the late summer, 10-DAT and BAC were also found to accumulate in the phloem region in both seasons, but the distribution patterns seemed to have no significant difference between the two seasons (Supplementary Figures S2C, D). CE, EDT, and paclitaxel were suggested to have a similar distribution in each season, but the patterns were found to differ between seasons. Their main distribution was found in the periderm and the earlier-forming phloem in the late summer, then shifted to the later-forming phloem and the cambium in the spring (Supplementary Figures S2E–G). As for 7-epi-taxol, the highest content was detected in the periderm in both seasons, with no significant difference in its distribution pattern (Supplementary Figure S2H).

**TABLE 1:** Rough tissue assignment of the tangential sections for quantification.

Section number	Distance from rhytidome/mm	Tissue assignment
1	0	Rhytidome, periderm
2	0.1	Periderm, phloem
3–7	0.2–0.6	Phloem
8–9	0.7–0.8	Later- (Latest-) forming phloem
10	0.9	Cambium
11	1	Cambium, latest-forming xylem
12	1.1	Latest-forming xylem

**FIGURE 4:**
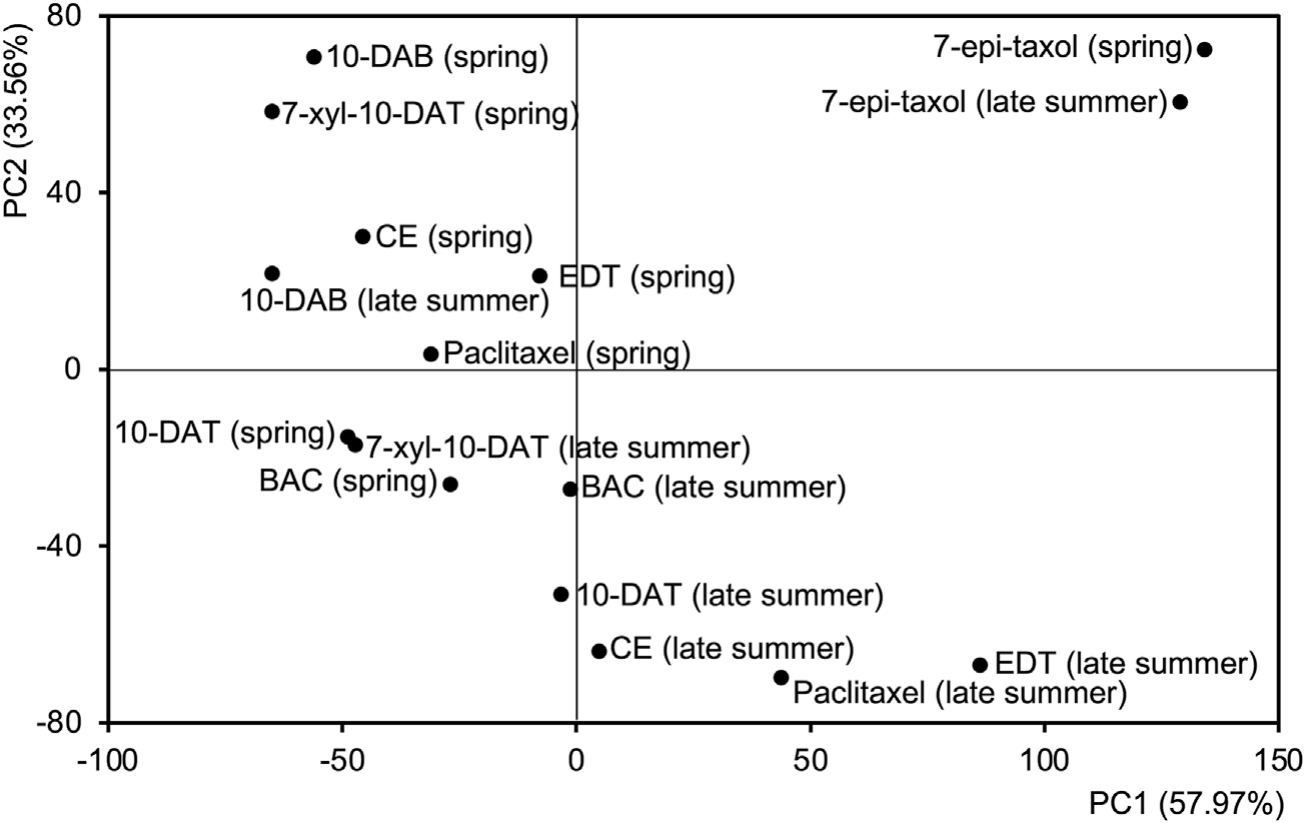
Principal component analysis (PCA) based on the radial content of eight taxanes of the tangential sections from late summer and spring. The first two principal components are shown.

A principal component analysis (PCA) was further achieved to separate the distribution patterns of taxanes in different seasons (Figure 4). PC1 and PC2 explained 57.97% and 33.56% of the difference, respectively, dividing the taxane distribution patterns into four categories, which suggested the similarities of taxane distributions in different seasons. The distributions of 10-DAB in both seasons and 7-xyl-10-DAT, CE, EDT, paclitaxel in spring were classified together, with their accumulation at the later-forming phloem. Differently, in the late summer, the distribution of 7-xyl-10-DAT was grouped with 10-DAT and BAC in both seasons, which corresponded to their peak content in phloem; also, another distinguished grouping of the distributions of CE, EDT, and paclitaxel in the late summer suggested their shift between seasons. As the fourth category, the distribution patterns of 7-epi-taxol were separated from the other seven taxanes, which was consistent with their unique distribution pattern.

### 2.2 Cryo-TOF-SIMS spectra of taxane standards and the freeze-fixed transverse surface of the *T. cuspidata* stem

**FIGURE 5:**
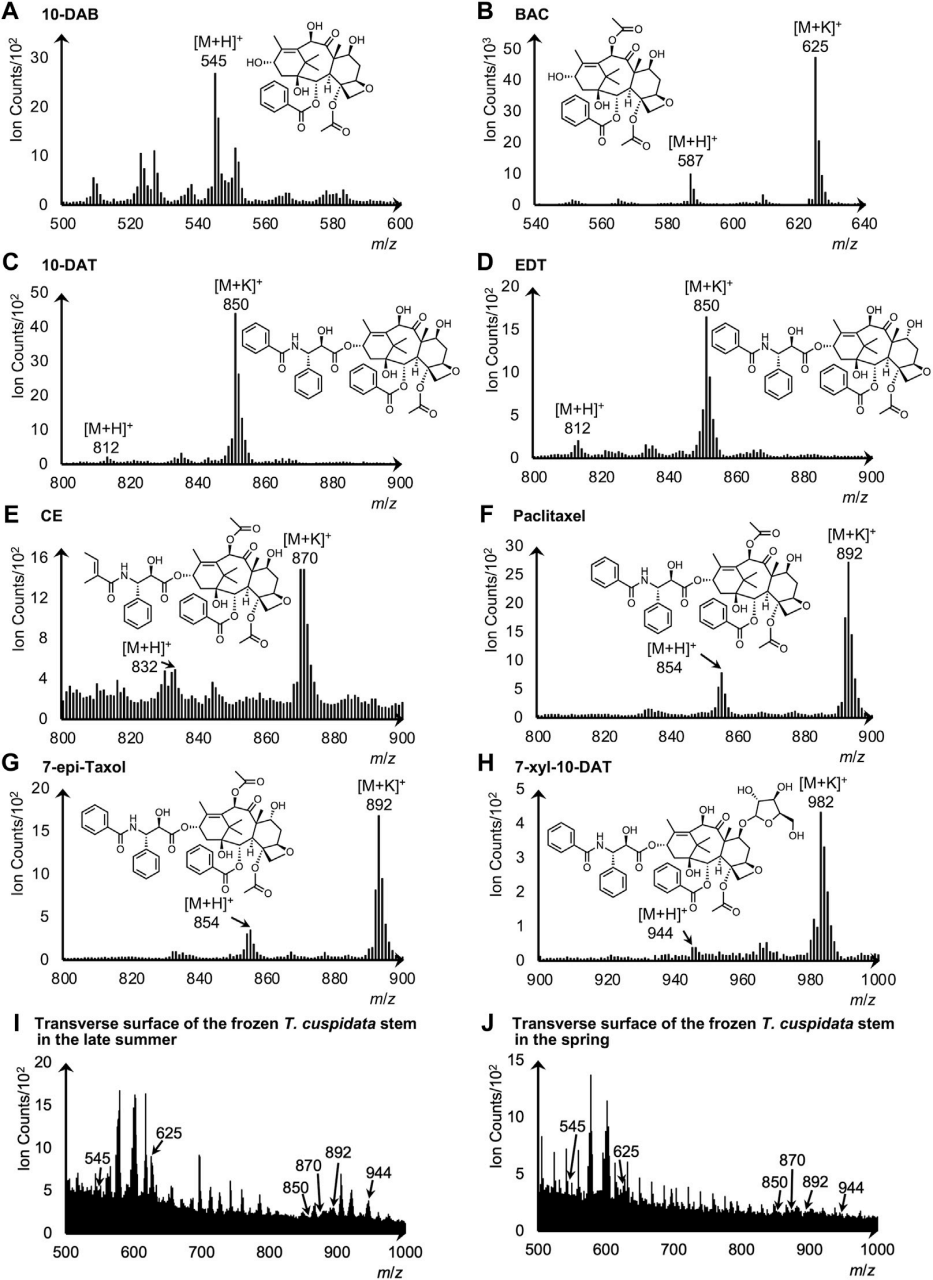
Cryo-TOF-SIMS spectra and chemical structures of **(A)** 10-DAB, **(B)** BAC, **(C)** 10-DAT, **(D)** EDT, **(E)** CE, **(F)** paclitaxel, **(G)** 7-epi-taxol, and **(H)** 7-xyl-10-DAT. Cryo-TOF-SIMS spectra obtained from the frozen, hydrated transverse surface of *T. cuspidata* stems from **(I)** late summer and **(J)** spring in the phloem region. Standard chemicals of taxanes were dissolved at *ca.* 50 mM in 80% EtOH and frozen for measurements.

Standard chemicals of the eight taxanes were measured by cryo-TOF-SIMS to determine their characteristic secondary ions. The acquired standard spectra are shown together with two typical spectra obtained from the transverse surface of freeze-fixed *T. cuspidata* stems in the late summer and the spring in Figure 5, and the masses with errors of the detected ions are shown in Supplementary Table S2.

For 10-DAB, the protonated molecular ion [M + H]^+^ at *m*/*z* 545 was detected at the highest intensity as the representative secondary ion (Figure 5A). While for BAC, 10-DAT, EDT, CE, paclitaxel, 7-epi-taxol, and 7-xyl-10-DAT, signals of [M + H]^+^ and the potassium adducts [M + K]^+^ were both detected (Figures 5B–H), and thus served as the candidates of the characteristic secondary ions.

Cryo-TOF-SIMS spectra acquired from the surface of *T. cuspidata* samples in the late summer and the spring (Figures 5I, J) exhibited the signals of characteristic ions of taxanes. Consistent with the standard spectrum, the secondary ion of 10-DAB was detected at *m*/*z* 545. For the other seven taxanes, the candidate ions of stronger detection were determined as representative secondary ions, which were the [M + K]^+^ ions of BAC, 10-DAT, EDT, CE, paclitaxel, 7-epi-taxol and the [M + H]^+^ ion of 7-xyl-10-DAT. Therefore, in the spectra of the *T. cuspidata* samples from both seasons, the signals of BAC, CE, 7-xyl-10-DAT, as well as the overlapping signals of 10-DAT and EDT, paclitaxel and 7-epi-taxol, were recognized at *m*/*z* 625, 870, 944, 850, and 892, respectively.

### 2.3 The distribution patterns of taxanes in the freeze-fixed *T. cuspidata* stems from late summer and spring

**FIGURE 6:**
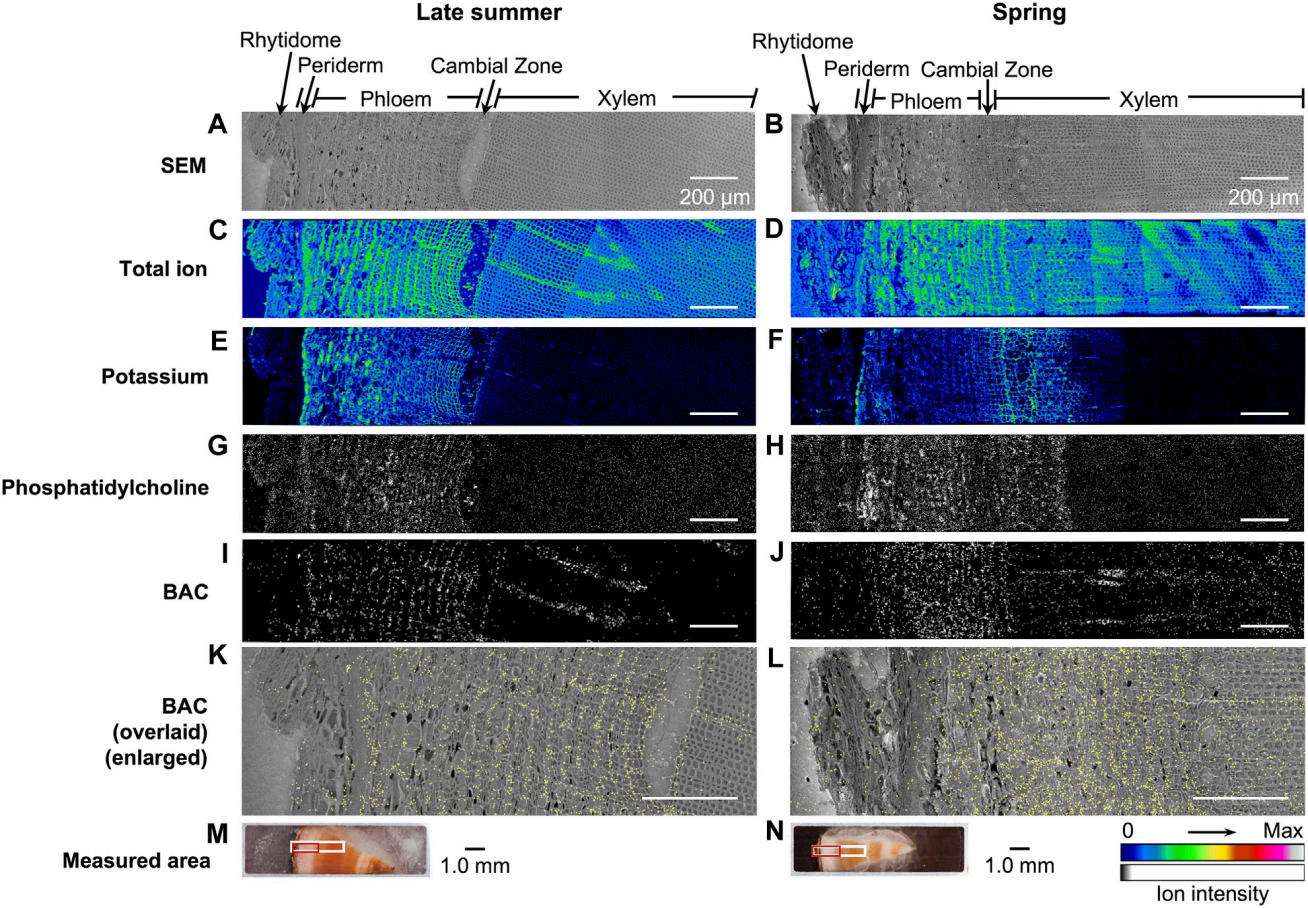
Cryo-TOF-SIMS/SEM results and microscopic images of freeze-fixed stems of *T. cuspidata* at the transverse surface. Cryo-SEM images of the freeze-etched sample in **(A)** late summer and **(B)** spring after cryo-TOF-SIMS analysis. Cryo-TOF-SIMS images for positive ions of total ion of **(C)** the late summer sample and **(D)** the spring sample, potassium at *m*/*z* 38.96 of **(E)** the late summer sample and **(F)** the spring sample, phosphatidylcholine at *m*/*z* 184 of **(G)** the late summer sample and **(H)** the spring sample, BAC at *m*/*z* 625 of **(I)** the late summer sample and **(J)** the spring sample, and the overlay images of BAC ion (yellow) on the enlarged cryo-SEM image of **(K)** the late summer sample and **(L)** the spring sample illustrating the tissue-specific distribution. Optical microscopy images of the **(M)** late summer and **(N)** spring *T. cuspidata* stem blocks set in the sample holder showing the regions measured by cryo-TOF-SIMS/SEM [marked in white for **(A–J)**, in red for **(K,L)**]. Scale bars are 200 μm for **(A–L)** and 1.0 mm for **(M–N)**.

The distribution of taxanes in the freeze-fixed stems of *T. cuspidata* from the late summer and the spring is shown in Figure 6; Supplementary Figures S3, S4. *T. cuspidata* sample blocks from the two seasons were firstly measured by cryo-TOF-SIMS (Figures 6C–J; Supplementary Figures S3D–K, S4D–K, with the line scan results of ion counts of the same surfaces) from rhytidome to xylem (Supplementary Figures S3L, S4L), then transferred to cryo-SEM for conducting observation (Figures 6A, B; Supplementary Figures S3B, S4B) of the same measurement area after appropriate freeze-etching for contrast enhancing of the cryo-SEM images (Supplementary Figure S5). To further assign the detailed tissue structures, the transverse surface sample sections were obtained from the same sample blocks analyzed by cryo-TOF-SIMS/SEM, stained by toluidine blue, and observed by optical microscopy (Supplementary Figures S3A, S4A). Additionally, the distribution of BAC, a representative taxane detected at relatively higher intensity, was overlaid on the SEM image for detailed discussion at the cellular level (Figure 6).

Potassium (*m*/*z* 38.96), as the most abundant inorganic cation in plants (Wang et al., 2013), was reported to be strongly detected in the plant cells with biological activities in TOF-SIMS. In the TOF-SIMS spectra obtained from the transverse surface of *T. cuspidata* stems from both seasons (Figures 5I, J), most target taxanes were detected in TOF-SIMS as potassium adduct ions [M + K]^+^. Also, potassium was specifically detected in the periderm, phloem, cambial zone, and specific structures in the xylem at higher intensity (Figures 6E, F; Supplementary Figures S3D, S4D), which tended to be similar to the position of living cells. In addition, phosphatidylcholine suggests the biological activity of cells as a principal constituent of cell membranes. A fragment ion of phosphatidylcholine, phosphocholine ion ([C_5_H_15_NO_4_P]^+^) at *m*/*z* 184, was reported as a characteristic secondary ion for the mapping of phosphatidylcholine by TOF-SIMS (Okumura et al., 2017; Gong et al., 2023). Therefore, the cryo-TOF-SIMS images of the ions at *m*/*z* 184 for the mapping of phosphatidylcholine are also displayed to represent the locations of living cells (Figure 6G, H; Supplementary Figures S3E, S4E).

Line scans showed the ion counts in the measured areas (Supplementary Figures S3D–K, S4D–K), indicating the major distribution of taxanes in the phloem and the differences in their accumulation at different sites between seasons, which corresponded with the radial quantitative distributions discussed in 2.1 to some extent. However, the intensity of secondary ion detection in the resulting images for most of the target taxanes was considered insufficient to achieve further discussion at the cellular level. Therefore, BAC, as the representative taxane in this study and a significant intermediate involved in the biosynthesis of paclitaxel, was discussed in detail in its tissue-specific distribution (Figure 6). Corresponding with the quantification results by LC-MS, BAC was accumulated in the phloem region and was detected around the cambial zone at a lower intensity. In conjunction with the SEM images (Figures 6K, L), such distribution was considered to be specifically in the sieve cells in the phloem, the cambium, and the ray parenchyma cells in the latest-forming xylem on the measured surfaces (Figures 6M, N). It may be noticed that the ion intensity of BAC showed stronger detection by TOF-SIMS in the spring sample than in the late summer sample, which was contrary to the quantification results discussed in 2.1. This might be due to the unavoidable matrix effect by the other chemical components co-existing in the plant sample, which may partly lead to the weak detection of other taxanes as well.

## 3 Discussion

So far, the variation in the concentration of several taxanes among seasons has been reported in *T. baccata* (Glowniak et al., 1999) and *T. brevifolia* (Vance et al., 1994). Also, MALDI-MS imaging has been applied for the visualization of taxanes in *T*. *media* (Soliman & Raizada, 2020; Yu et al., 2020) and *T*. *mairei* (Yu et al., 2023), which suggested specific distribution of taxanes in different major tissues. Such works brought the possibility for the overall study of the taxanes in *Taxus* species; however, detailed information on the taxane distribution among seasons, as well as at the cellular level in *T. cuspidata* is still limited.

Quantitative analysis of tangential sections from the rhytidome to the latest-forming xylem was achieved to investigate the seasonal difference of the distribution of eight taxanes in the stem of *T. cuspidata*. The overall content of taxanes was generally higher in the late summer than in the spring. It was reported that *Taxus* plants tended to maintain higher levels of carbohydrates during the non-growing season, which was associated with a lower plant growth rate, to cope with unfavorable conditions and improve survivability (Pan et al., 2024). Therefore, it may be speculated that the late-summer *T. cuspidata* accumulated more bioactive taxanes towards the upcoming dormancy in fall and winter to enhance its resistance during the dormant period. In this study, taxanes distributed in the periderm and earlier-forming phloem were suggested to have higher content in the late summer and lower in the spring. Considering the potential purpose of the plant increasing its competitiveness, taxane distribution at the outer side of the *T. cuspidata* stem may confirm their proposed defensive roles *in planta* (Manz et al., 2022). Regarding the taxane distribution in the outermost rhytidome, it was detected at low intensity by LC-MS and cryo-TOF-SIMS analyses; however, since the outermost cell layers were fragmented as observed and thinner than 100 μm, this time the distribution in the rhytidome cannot be solidly confirmed. It was reported that paclitaxel was stored in and released from the dead bark, possibly for defense purposes (Vance et al., 1994; Soliman & Raizada, 2020), so it can be supposed that taxanes did exist in the rhytidome, although further confirmation is still needed. One alternative reason for the relatively lower taxane content in spring should be their possible utilizations. Peak contents of taxanes, including CE, EDT, and paclitaxel, were observed around the cambial zone in spring, which is considered to be related to some special physiological activities of the plant, e.g., the activated cell differentiation during the germination period. However, since the distribution patterns and amounts of taxanes are under explored in the intermediate period covering the plant dormancy between our two samplings, how the taxanes were involved in the plant physiological processes under the potential seasonal impact components remains to be investigated.

Further, as one of the most important intermediates involved in the biosynthesis of paclitaxel, the specific distribution of BAC at the cellular level was discussed by the cryo-TOF-SIMS analysis and the tissue assignment by cryo-SEM analysis and optical microscopy. BAC was considered to be distributed in the phloem sieve cells and the ray parenchyma cells in the latest-forming xylem in the stem of *T. cuspidata*. These tissues carry out radial and axial transport of functional components in the plant stem (Aoki et al., 2017). Thus, the suggested distribution pattern of BAC was thought to be related to its transport. Further tissue-specific localization at protein or gene levels on the stem of *T. cuspidata* is required to clarify the relations between transportation and the sites for taxanes involving biosynthesis.

## 4 Materials and methods

### 4.1 Plant materials

**FIGURE 7:**
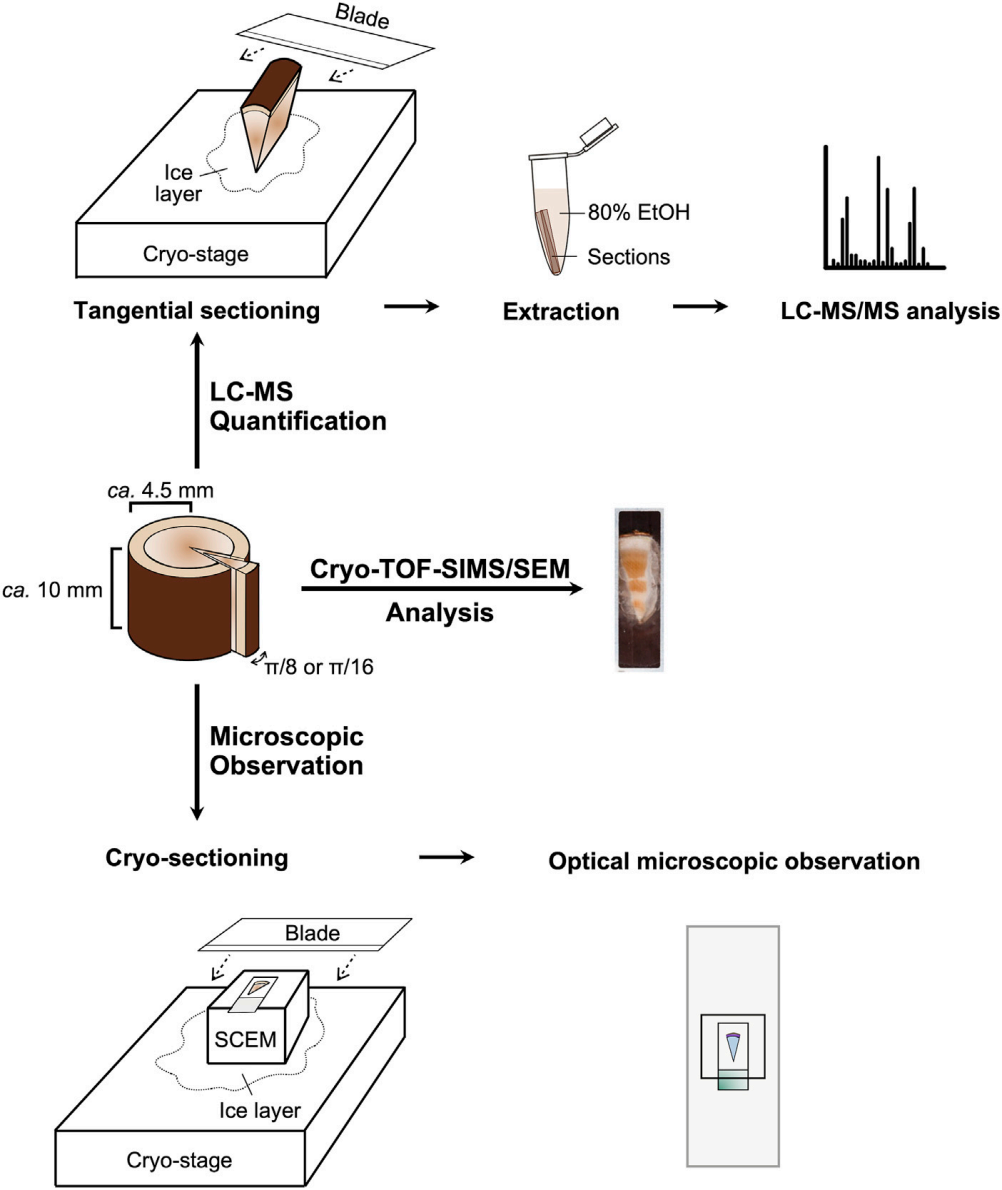
Experiment scheme.

Plant sampling was achieved on 20 September 2022 in the late summer and 26 May 2023 in the spring. Each sample disk (thickness of *ca.* 10 mm) was obtained from *T. cuspidata* trees (3.5–4.5 m in height, five- to ten-year-old branches) grown in the Inabu Field (Toyota, Japan) affiliated to the Graduate School of Bioagricultural Sciences, Nagoya University and cut into small blocks (circular sector with a radius of *ca.* 4.5 mm and central angle of π/8 or π/16) containing bark and wood. The blocks were quick-frozen with liquid Freon^®^ 22 (DuPont) at −160°C and stored at −80°C. Details of the sampling procedure are illustrated in Figure 7.

### 4.2 Chemicals and reagents

Standard chemicals including paclitaxel (FUJIFILM Wako Pure Chemical Corp., Osaka, Japan), 10-DAB (Tokyo Chemical Industry Co., Ltd., Tokyo, Japan), BAC and CE (Selleck Chemicals LLC., TX, United States), 7-xyl-10-DAT, 7-epi-taxol, EDT, and 10-DAT (MedChem Express LLC., NJ, United States) were purchased and used as received. EtOH, acetonitrile (ACN), and formic acid (FA) of LC-MS grade were purchased from FUJIFILM Wako Pure Chemical Corp. The ultra-pure water for LC-MS measurements was obtained from a Milli-Q water purification system (Millipore, MA, United States).

### 4.3 LC-MS measurements

Frozen sample blocks of *T. cuspidata* stem were cut from the rhytidome to the latest-forming xylem into 12 serial tangential sections of 100-μm thickness with a sliding microtome (REM-710, Yamato Kohki Industrial Co., Ltd., Asaka, Japan). Each section was extracted with 1 mL 80% EtOH aq. at 60°C with ultrasonic assisting for 1 hour, dried by SpeedVac (Thermo Scientific, MA, United States) centrifugation, and re-dissolved in 50 μL 80% EtOH aq. for analysis.

LC-MS/MS measurements of the extracts were achieved with Thermo Scientific Vanquish UHPLC-Orbitrap Exploris 240 equipped with an Accucore Vanquish C18+ UPLC column (100 × 2.1 mm, C_18_, 1.5 μm, Thermo Scientific). Target compounds were separated by a binary buffer system of 0.1% (*v*/*v*) FA aq. (buffer A) and 0.1% (*v*/*v*) FA in ACN (buffer B) at a flow rate of 0.4 mL/min. The gradient for *T. cuspidata* extract analysis was 10 min in total and set as follows: hold at 40% (*v*/*v*) buffer B in 2 min, from 40% to 55% (*v*/*v*) buffer B in 5 min, from 55% to 90% (*v*/*v*) buffer B in 1 min, holding at 90% (*v*/*v*) buffer B for 1 min, declining to 40% (*v*/*v*) buffer B in 0.1 min, and holding at 40% (*v*/*v*) buffer B for 0.9 min. All the chromatograms were taken at column temperature 30°C. Full MS scans were performed in the Orbitrap mass analyzer over an *m*/*z* range of 200–1,200 with a mass resolution of 60,000. Tandem MS was performed using an isolation window of *m*/*z* 2 and HCD fragmentation with normalized collision energies of 30. The measurements were carried out in three replicates using three blocks from the same sample disk to evaluate the average amount and standard errors. PCA data processing was performed on Python (3.10.14) with Spyder IDE (5.5.1) using scikit-learn library (1.4.2) (Pedregosa et al., 2011).

### 4.4 Cryo-TOF-SIMS/SEM analyses

Details of the manufactured cryo-TOF-SIMS/SEM system were described previously (Kuroda et al., 2013; Masumi et al., 2014). For each of the late summer and spring seasons, one frozen sample block was fixed in a copper holder by ice embedding and cut in the glove box under a dry N_2_ environment (sample temperature below −30°C) to achieve a clean and flat surface, then introduced to the cryo-TOF-SIMS system by a cryo-vacuum shuttle for analysis. Positive ion images were obtained by cryo-TOF-SIMS (TRIFT-III spectrometer, ULVAC-PHI Inc., Chigasaki, Japan). 22 keV Au_1_^+^ at the current of 27 nA was used as the primary ion beam, and a low-energy pulsed electron gun (30.0 eV) was used for surface charge compensation. Other conditions were set as follows: raster size at 400 × 400 μm, measurement time of 20–30 min, pulse width at 13 ns (non-bunched, image) or 1.9 ns(bunched, spectrum), spot size at 1.0 μm (image), the temperature at −120 to −130°C, vacuum level below 1.0 × 10^−7^ Pa. Standard chemicals of target compounds were dissolved in 80% EtOH at *ca.* 50 mM, dropped on the achieved smooth, flat ice surfaces (made up of 50 mM KCl solution) and dried up, then measured by cryo-TOF-SIMS in the same procedure in bunched mode.

After cryo-TOF-SIMS measurements, the sample block was transferred to cryo-SEM (S-3400N, Hitachi High-Tech Corp., Tokyo, Japan) by a cryo-vacuum shuttle. To enhance the contrast of SEM images, the frozen and hydrated sample surface was freeze-etched at −90°C, then observed at around −110°C to obtain images of the same region measured by cryo-TOF-SIMS. The acceleration voltage was set at 1.5 kV, and the working distance was 10 mm.

Obtained cryo-TOF-SIMS images were connected using WinCadence 5.1.2.8 (ULVAC-PHI Inc.) and MATLAB R2022a (The MathWorks Inc., MA, United States) with PLS Toolbox 8.8.1 (Eigenvector Research Inc., WA, United States) without any ion count normalization. Color scales of images were adjusted using ImageJ software (The National Institutes of Health, MD, United States) (Schneider et al., 2012). The SEM images and the overlay images of the selected target ion on the SEM images were prepared using Photoshop CS5 Extended (Adobe Systems Inc., CA, United States).

### 4.5 Cryo-sectioning and microscopic observations

Cryo-sectioning was conducted at the transverse surface following a modified procedure based on Kawamoto’s film method (Kawamoto, 2003; Kawamoto et al., 2021) (Supplementary Figure S6). First, frozen blocks after the cryo-TOF-SIMS/SEM analyses were immersed in the embedding medium (SCEM, SECTION-LAB Co., Ltd., Yokohama, Japan) at room temperature for 30 min. After thawing, the blocks were embedded in SCEM and cut into sections at 2-μm thickness with a sliding microtome. Obtained sections were pretreated before staining by the following steps: thawed at room temperature for 20 s, submerged three times in EtOH for 10 s, 10 s, 20 s, submerged in 4% paraformaldehyde solution for 5 min, rinsed under running water for 10 min. After rinsing, the sections were stained with toluidine blue and observed with an optical microscope (BX-60, Olympus Corp., Hachioji, Japan).

## 5 Conclusion

In this study, the radial distribution of eight target taxanes including paclitaxel, 10-DAB, BAC, CE, 7-xyl-10-DAT, 7-epi-taxol, EDT, and 10-DAT have been discussed in the transverse surface of freeze-fixed *T. cuspidata* stems from late summer and spring seasons by cryo-TOF-SIMS/SEM visualization and quantitative LC-MS analysis. The overall amount of taxanes was higher in the late summer than in the spring. Their radial distribution was generally found at higher concentration in the phloem, the cambium and lower level in the periderm, the latest-forming xylem, with different taxanes showing several patterns with distinction between seasons, which were considered related to seasonal physiological behaviors of *T. cuspidata*. In addition, the distribution of BAC was investigated at the cellular level, which was regarded in the sieve cells of phloem, the cambium cells, and the ray parenchyma cells in the latest-forming xylem and suggested its transport in the radial and axial directions in the *T. cuspidata* stem. Characterizing the microscopic distribution of taxanes in the *T. cuspidata* stem is expected to play a role in the further study of their biosynthesis and *in planta* behaviors.

## Data Availability

The original contributions presented in the study are included in the article/Supplementary Material, further inquiries can be directed to the corresponding author.
