# Supplementary material for: Microscopic distribution of taxanes in freeze-fixed stems of Taxus cuspidata
Source: Front Chem. 2024 Aug 12;12:1437141. doi: 10.3389/fchem.2024.1437141 (PMC11345223; doi:10.3389/fchem.2024.1437141)
Supplement: Supplementary file 1 [file DataSheet1.DOCX]

Supplementary Material

Microscopic Distribution of Taxanes in Freeze-fixed Stems of *Taxus cuspidata*

Qinyue Gong^1^, Dan Aoki^1^*, Masato Yoshida^1^, Kazuhiko Fukushima^1^

^1^Graduate School of Bioagricultural Sciences, Nagoya University, Nagoya, Japan

*** Correspondence:** Corresponding Author: Dan Aoki (aoki.dan@nagoya-u.jp)

Keywords:

*Taxus cuspidata*, *Taxaceae*, cryo-TOF-SIMS/SEM, mass spectrometry imaging, taxanes, paclitaxel

# Supplementary Figures and Tables

**Figure S1.** MS^2^ spectra obtained by LC-MS/MS analysis of the *T. cuspidata* sample and standard solutions of the eight taxanes under the same quantitative conditions.

**Table S1.** Qualitative analysis of taxanes in *T. cuspidata* by LC-MS/MS.

**Figure S2.** Radial distribution of the eight taxanes by LC-MS/MS.

**Table S2.** Qualitative analysis of taxanes in *T. cuspidata* by TOF-SIMS.

**Figure S3.** Cryo-TOF-SIMS/SEM analysis results and microscopic images of the *T. cuspidata* stem from the late summer at the transverse surface.

**Figure S4.** Cryo-TOF-SIMS/SEM analysis results and microscopic images of the *T. cuspidata* stem from the spring at the transverse surface.

**Figure S5.** Cryo-SEM images of the freeze-fixed stems of *T. cuspidata* in the late summer and the spring before and after freeze-etching, at the transverse surface.

**Figure S6.** Modified procedure of section preparation for optical microscopic observation based on Kawamoto’s film method.

**Figure S1.** MS^2^ spectra obtained by LC-MS/MS analysis of the *T. cuspidata* sample and standard solutions of the eight taxanes under the same quantitative conditions.

(**Figure S1.** continued)

(**Figure S1.** continued)

| Compound | Chemical  formula | Ion  species | Expected  *m*/*z* | Observed  *m*/*z* | Average error (ppm) | Fragment  ions |
| --- | --- | --- | --- | --- | --- | --- |
| 10-DAB | C_29_H_36_O_10_ | [M+H]^+^ | 545.2383 | 545.2371 | −2.2009 | 527.2290 [M+H−H_2_O]^+^,  363.1805 [M+H−CH_3_COOH−PhCOOH]^+^,  345.1688 [M+H−CH_3_COOH−PhCOOH−H_2_O]^+^,  327.1592 [M+H−CH_3_COOH−PhCOOH−2H_2_O]^+^,  309.1487 [M+H−CH_3_COOH−PhCOOH−3H_2_O]^+^ |
| BAC | C_31_H_38_O_11_ | [M+H]^+^ | 587.2489 | 587.2474 | −2.5543 | 405.1906 [M+H−CH_3_COOH−PhCOOH]^+^,  387.1798 [M+H−CH_3_COOH−PhCOOH−H_2_O]^+^,  345.1696 [M+H−2CH_3_COOH−PhCOOH]^+^,  327.1591 [M+H−2CH_3_COOH−PhCOOH−H_2_O]^+^,  309.1483 [M+H−2CH_3_COOH−PhCOOH−2H_2_O]^+^ |
| 10-DAT | C_45_H_49_NO_13_ | [M+H]^+^ | 812.3278 | 812.3260 | −2.2159 | 527.2292 [M+H−C_16_H_15_NO_4_]^+^,  286.1072 [M+H−C_29_H_34_O_9_]^+^ |
| EDT | C_45_H_49_NO_13_ | [M+H]^+^ | 812.3278 | 812.3268 | −1.2310 | 286.1071 [M+H−C_29_H_34_O_9_]^+^ |
| CE | C_45_H_53_NO_14_ | [M+H]^+^ | 832.3539 | 832.3525 | −1.6820 | 754.3195 [M+H−H_2_O−CH_3_COOH]^+^,  569.2383 [M+H−C_14_H_17_NO_4_]^+^,  509.2171 [M+H−C_14_H_17_NO_4_−CH_3_COOH]^+^,  264.1230 [M+H−C_31_H_36_O_10_]^+^ |
| Paclitaxel | C_47_H_51_NO_14_ | [M+H]^+^ | 854.3383 | 854.3367 | −1.8728 | 569.2351 [M+H−C_16_H_15_NO_4_]^+^,  509.2167 [M+H−C_16_H_15_NO_4_−CH_3_COOH]^+^,  449.1953 [M+H−C_16_H_15_NO_4_−2CH_3_COOH]^+^,  387.1797 [M+H−C_16_H_15_NO_4_−PhCOOH−CH_3_COOH]^+^,  327.1592 [M+H−C_16_H_15_NO_4_−PhCOOH−2CH_3_COOH]^+^,  286.1074 [M+H−C_31_H_36_O_10_]^+^ |
| 7-Epi-taxol | C_47_H_51_NO_14_ | [M+H]^+^ | 854.3383 | 854.3371 | −1.4046 | 509.2170 [M+H−C_16_H_15_NO_4_−CH_3_COOH]^+^,  327.1592 [M+H−C_16_H_15_NO_4_−PhCOOH−2CH_3_COOH]^+^,  286.1075 [M+H−C_31_H_36_O_10_]^+^ |
| 7-Xyl-10-DAT | C_50_H_57_NO_17_ | [M+H]^+^ | 944.3699 | 944.3684 | −1.5884 | 659.2700 [M+H−C_16_H_15_NO_4_]^+^,  509.2166 [M+H−C_16_H_15_NO_4_−C_5_H_10_O_5_]^+^,  286.1074 [M+H−C_34_H_42_O_13_]^+^ |

**Table S1.** Qualitative analysis of taxanes in *T. cuspidata* by LC-MS/MS*.*

**
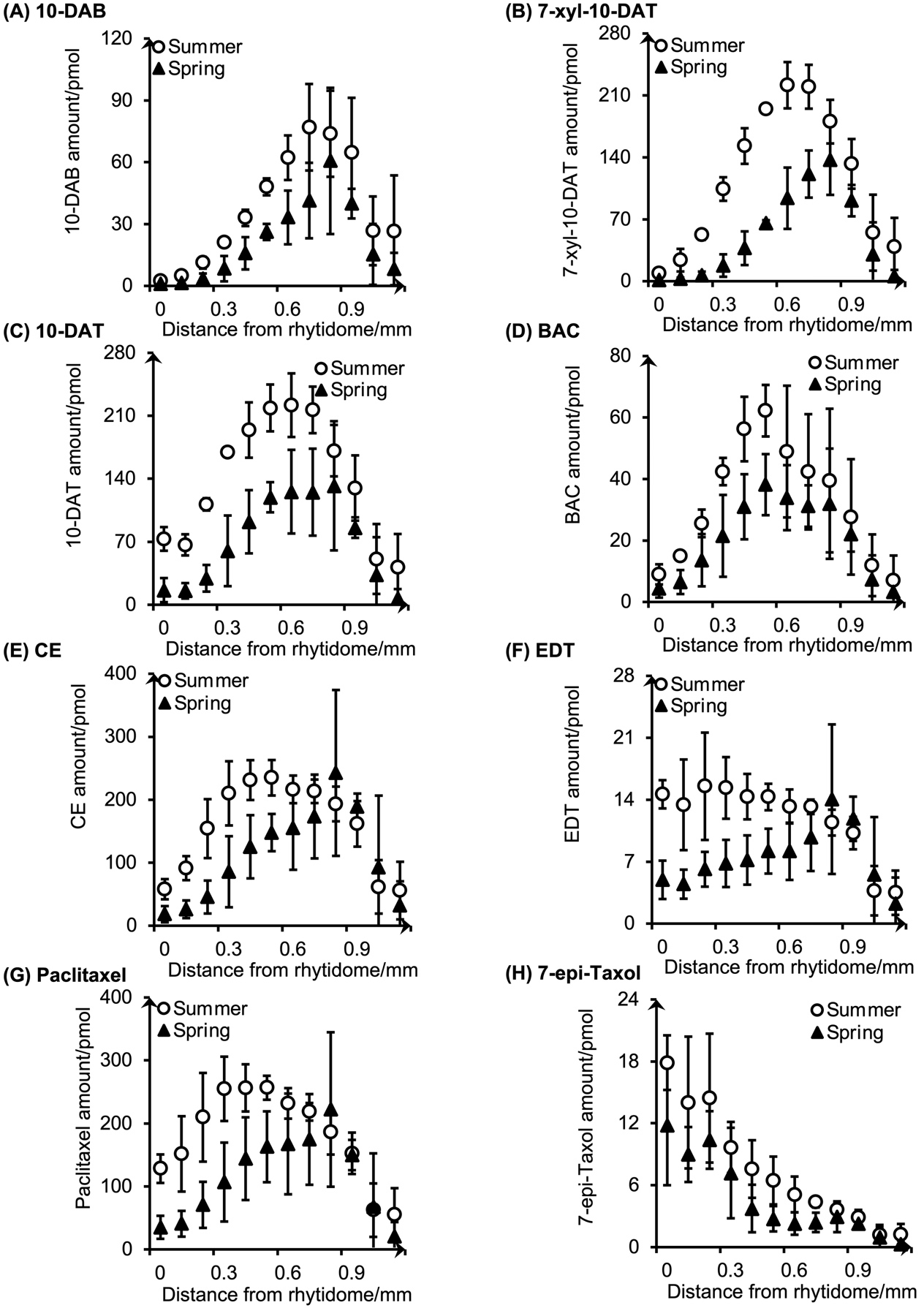
Figure S2.** Radial distributions of (A) 10-DAB, (B) 7-xyl-10-DAT, (C) 10-DAT, (D) BAC, (E) CE, (F) EDT, (G) paclitaxel, and (H) 7-epi-taxol in the late summer and spring *T. cuspidata* stems, quantified by LC-MS/MS using serial tangential sections. Means and standard deviations for each sample were obtained from three sets of measurements (n = 3) using different sample blocks from the same disk.

| Compound | Chemical formula | Ion species | Expected *m*/*z* | Observed *m*/*z* | Error (ppm) |
| --- | --- | --- | --- | --- | --- |
| 10-DAB | C_29_H_36_O_10_ | [M+H]^+^ | 545.2383 | 545.2940 | 102.1572 |
| BAC | C_31_H_38_O_11_ | [M+K]^+^ | 625.2048 | 625.2718 | 107.1649 |
| 10-DAT | C_45_H_49_NO_13_ | [M+K]^+^ | 850.2837 | 850.2764 | −8.5854 |
| EDT | C_45_H_49_NO_13_ | [M+K]^+^ | 850.2837 | 850.2424 | −48.5720 |
| CE | C_45_H_53_NO_14_ | [M+K]^+^ | 870.3098 | 870.2805 | −33.6662 |
| Paclitaxel | C_47_H_51_NO_14_ | [M+K]^+^ | 892.2942 | 892.2647 | −33.0608 |
| 7-Epi-taxol | C_47_H_51_NO_14_ | [M+K]^+^ | 892.2942 | 892.2858 | −9.4139 |
| 7-Xyl-10-DAT | C_50_H_57_NO_17_ | [M+H]^+^ | 944.3699 | 944.6237 | 268.7506 |

**Table S2.** Qualitative analysis of taxanes in *T. cuspidata* by TOF-SIMS*.*

**
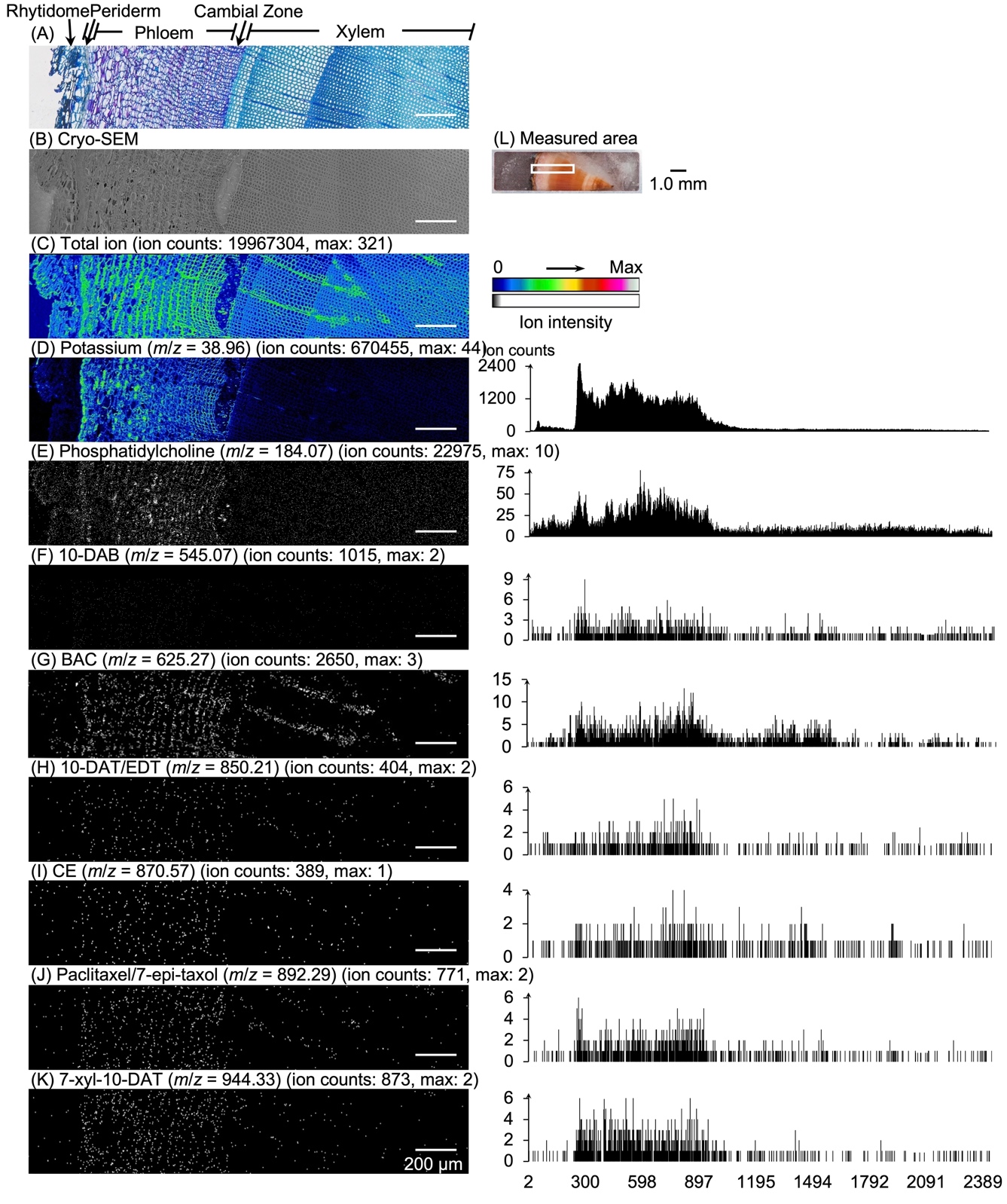
Figure S3.** Cryo-TOF-SIMS/SEM results and microscopic images of a freeze-fixed stem of *T. cuspidata* sampled in the late summer at the transverse surface. (A) Optical microscopic image of a toluidine blue stained section showing rhytidome, periderm, phloem, cambial zone, and xylem. (B) Cryo-SEM image of the freeze-etched sample after cryo-TOF-SIMS analysis. Cryo-TOF-SIMS images for positive ions of (C) total ion, (D) potassium at *m*/*z* 38.96, (E) phosphatidylcholine at *m*/*z* 184.07, (F) 10-DAB at *m*/*z* 545.07, (G) BAC at *m*/*z* 625.27, (H) 10-DAT and EDT at *m*/*z* 850.21, (I) CE at *m*/*z* 870.57, (J) paclitaxel and 7-epi-taxol at *m*/*z* 892.29, (K) 7-xyl-10-DAT at *m*/*z* 944.33. Line scan results for ion counts of (D–K) are accordingly shown on the right. (L) Optical microscopy image of the *T. cuspidata* stem block set in the sample holder showing the area measured by cryo-TOF-SIMS/SEM (marked region). Scale bars are 200 μm for (A–K) and 1.0 mm for (L).

**
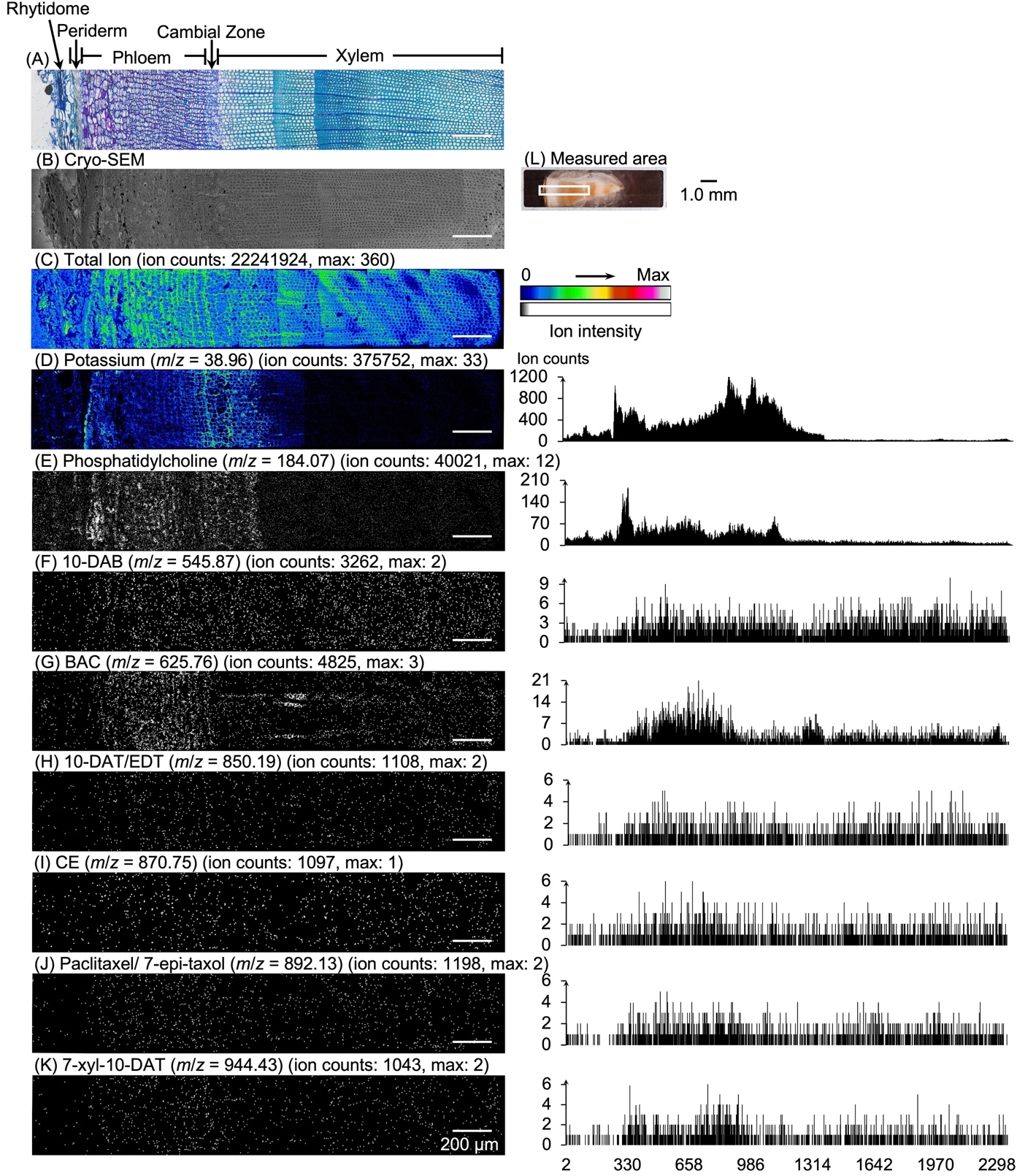
Figure S4.** Cryo-TOF-SIMS/SEM results and microscopic images of a freeze-fixed stem of *T. cuspidata* sampled in the spring at the transverse surface. (A) Optical microscopic image of a toluidine blue stained section showing rhytidome, periderm, phloem, cambial zone, and xylem. (B) Cryo-SEM image of the freeze-etched sample after cryo-TOF-SIMS analysis. Cryo-TOF-SIMS images for positive ions of (C) total ion, (D) potassium at *m*/*z* 38.96, (E) phosphatidylcholine at *m*/*z* 184.07, (F) 10-DAB at *m*/*z* 545.87, (G) BAC at *m*/*z* 625.76, (H) 10-DAT and EDT at *m*/*z* 850.19, (I) CE at *m*/*z* 870.75, (J) paclitaxel and 7-epi-taxol at *m*/*z* 892.13, (K) 7-xyl-10-DAT at *m*/*z* 944.43. Line scan results for ion counts of (D–K) are accordingly shown on the right. (L) Optical microscopy image of the *T. cuspidata* stem block set in the sample holder showing the area measured by cryo-TOF-SIMS/SEM (marked region). Scale bars are 200 μm for (A–K) and 1.0 mm for (L).

**
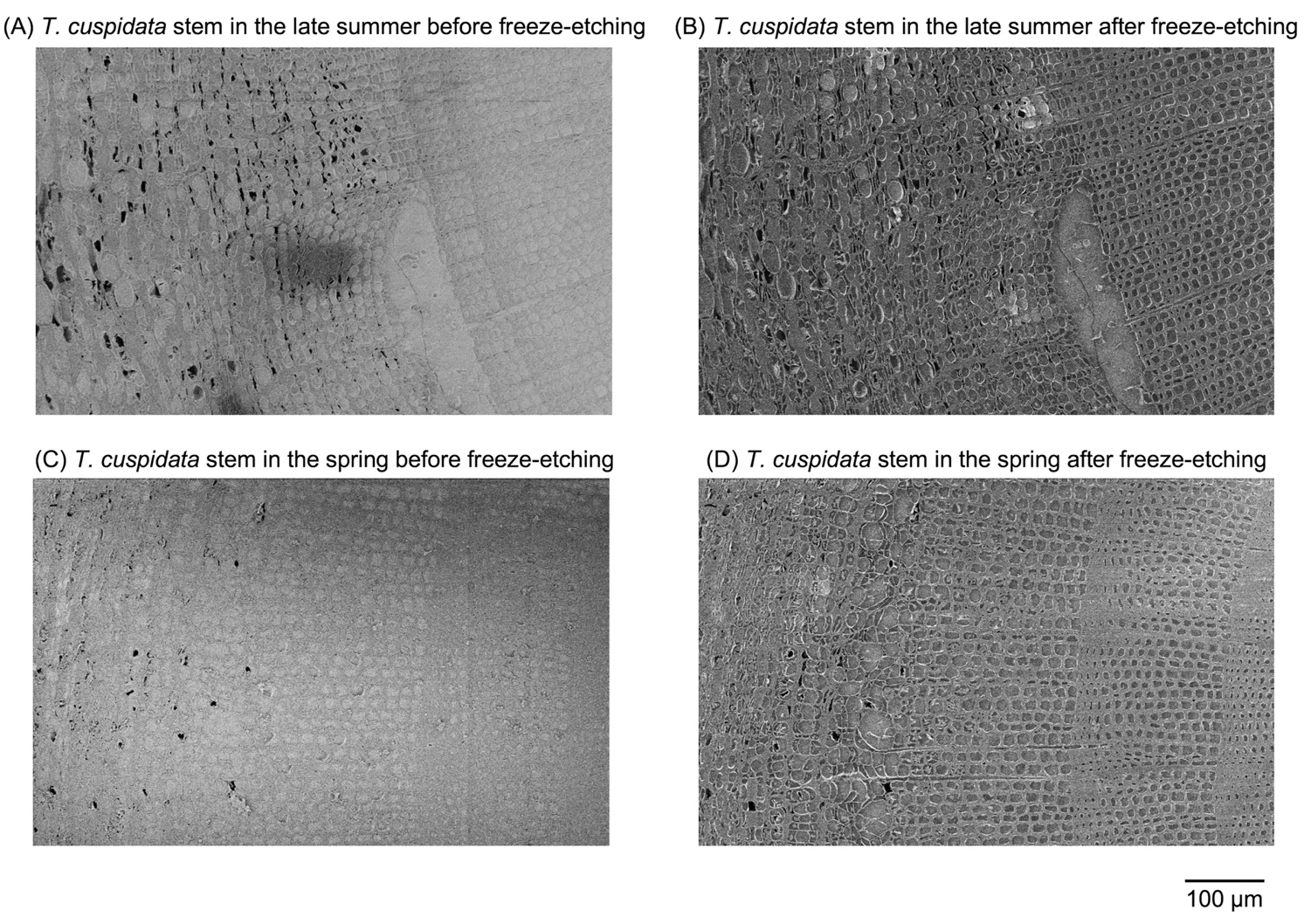
Figure S5.** Cryo-SEM images of freeze-fixed stems of *T. cuspidata* in the late summer (A) before and (B) after freeze-etching and in the spring (C) before and (D) after freeze-etching, at the transverse surface. Scale bar is 100 μm.

**Figure S6.** Modified procedure of section preparation for optical microscopic observation based on Kawamoto’s film method.
